# DING Proteins Extend to the Extremophilic World

**DOI:** 10.3390/ijms22042035

**Published:** 2021-02-18

**Authors:** Elena Porzio, Maria Rosaria Faraone Mennella, Giuseppe Manco

**Affiliations:** 1 Institute of Biochemistry and Cell Biology, CNR, Via P. Castellino 111, 80131 Naples, Italy; giuseppe.manco@cnr.it; 2 Department of Biology, Polytechnic School of Basic Sciences, University of Naples “Federico II”, 80126 Naples, Italy; faraone@unina.it

**Keywords:** DING protein, PARPSso, Archaea, prokaryotes, eukaryotes, extremophiles, phosphate-binding proteins

## Abstract

The DING proteins are ubiquitous in the three domains of life, from mesophiles to thermo- and hyperthermophiles. They belong to a family of more than sixty members and have a characteristic N-terminus, DINGGG, which is considered a “signature” of these proteins. Structurally, they share a highly conserved phosphate binding site, and a three dimensional organization resembling the “Venus Flytrap”, both reminding the ones of PstS proteins. They have unusually high sequence conservation, even between distantly related species. Nevertheless despite that the genomes of most of these species have been sequenced, the DING gene has not been reported for all the relative characterized DING proteins. Identity of known DING proteins has been confirmed immunologically and, in some cases, by N-terminal sequence analysis. Only a few of the DING proteins have been purified and biochemically characterized. DING proteins are heterogeneous for their wide range of biological activities and some show different activities not always correlated with each other. Most of them have been originally identified for different biological properties, or rather for binding to phosphate and also to other ligands. Their involvement in pathologies is described. This review is an update of the most recent findings on old and new DING proteins.

## 1. Introduction: The State of the Art of DING Proteins

The DING proteins are ubiquitous proteins (38–40 kDa), characterized by a conserved N-terminal sequence, DINGGG [1]. In the 1990s they were first identified in several animal and plant tissues, and then in most eukaryotes and a range of bacterial organisms [1].

The characteristic N-terminal amino acid signature D-I-N-G (for aspartate-isoleucine-asparagine-glycine) is highly conserved in proteins belonging to the superfamily of phosphate-binding proteins (PBP), as they possess a phosphate-binding pocket. Berna et al. [1] have originally defined the group of DING proteins as a family. However, the proteins are heterogeneous for their wide range of biological activities.

The most advanced studies of DING proteins up to 2013 were summarized by Bernier, who tried to classify these proteins on the basis of the N-terminal highly conserved sequence DINGG [2,3,4]. The choice of comparing their N-termini was due mainly to the lack of amino acid sequences for most of the DING family members.

Each of the five defined subclasses enclosed proteins even from different domains of life. This is an intriguing feature as in the same subclass are proteins exhibiting different biological activities and belonging to different species.

In the same year Sachdeva et al. (2013) [3] published a paper comparing the amino acid sequences of the most studied mesophilic DINGs from both prokaryotes and eukaryotes, highlighting both their common primary structure and basic similarity with PstS and alkaline phosphatase configuration (Venus flytrap model). This extends the structural features in common with different DING proteins.

Their roles are not completely clear yet; however, the ability to bind phosphate seems to be a common property, confirmed by three-dimensional structure analyses of both eukaryotic and prokaryotic DING proteins [1]. They are usually secreted, although some are found in the cytosol [4,5].

At the present, more than sixty DING proteins are known and occur widely in all the three domains of life, including Archaea [2,6,7,8,9]. There is increasing evidence that DING proteins are involved in many health-related processes as cancers and both bacterial (Pseudomonas) and viral (HIV) infections [1,3,10,11]. The few gene sequences available in databases are of great interest, because, despite the genomes of most of these species have been sequenced, the DING genes have not been reported for all the relative characterized DING proteins. This peculiar aspect is discussed in a separate section.

The identity of known DING proteins has been confirmed immunologically and, in some cases, by N-terminal sequence analysis [2,6,7,8,12]. Only a few DING proteins have been purified from various species and cell types, and biochemically characterized [2,6,7,8,9,12,13,14].

In line with the above overview of main features of DING proteins, we will present the state of the art of the DING proteins identified in Prokaryotes, Eukarya and extremophilic environment.

### 1.1. DING Proteins in Prokaryotes

In prokaryotes, DING proteins are part of a superfamily of PBP that comprises three subgroups, based on sequence similarities and functions: (1) ubiquitous membrane-bound or periplasmic “PstS proteins” that participate in uptake of phosphate and belong to the transmembrane ABC transporters. These periplasmic proteins do not have the characteristic N-terminal sequence, but show 20–25% of identity with the eukaryotic DING proteins; (2) some low relative molecular mass (*M_r_*) “alkaline phosphatases” only described in *Pseudomonas,* that are 40–50% identical to eukaryotic DINGs; and (3) “true bacterial DING proteins” that share around 70–80% identity with the eukaryotic ones [15].

All members of this superfamily show a common three-dimensional structure, comprising the conserved eight amino acid residues involved in phosphate binding [16,17].

In bacteria, PstS are involved in phosphate sensing and homeostasis, a phenomenon that seems to be involved in the virulence of some bacteria, in particular *Pseudomonas* genera that is a major pathogen for a wide range of eukaryotes, including vertebrates, non-vertebrates, and plants [18]. For examples, in *Pseudomonas aeruginosa* P14A, virulence is associated with the release of some enzymes, as alkaline phosphatase, that in association with membrane vesicles spread pathogenicity factors to epithelial cells during infection [19]. It has been reported that in low phosphate conditions, highly virulent multiantibiotic-resistant (MDR) strains of *P. aeruginosa* isolated from critically ill patients produce an abundance of PstS, located on extracellular finger-like structures, which contribute to bacterial adherence to intestinal epithelial cells [20]. In contrast to the previously described membrane vesicles, these appendages seem to remain associated with the bacterial surface, but it is possible that in both cases the presence of DING proteins is involved in conferring an adhesive and barrier disruptive phenotype against intestinal epithelial cells [20].

To do an upgrade of the already reported prokaryotic DING proteins [1,2,4,15] we recall the most well characterized ones and report the recently discovered DINGs.

Only one gene in prokaryotes, *Pflu*DING from *P. fluorescens* SBW25, has been cloned and expressed in *E. coli* [21]. The *Pflu*DING structure was determined and found to be closely similar to the “Venus flytrap” structure in which two globular domains hinge together to form the phosphate-binding site, with eight conserved residues H-bonded to phosphate, already seen in another human DING (in particular the human phosphate-binding protein (HPBP)) and in bacterial phosphate solute binding proteins (SBPs) [17,21]. It binds to a single phosphate ion, but has no detectable phosphatase activity (Table 1). It is an extracellular protein, whose expression is linked to low extracellular phosphate levels; this suggests that *Pflu*DING could be involved in extracellular scavenging of phosphates, which are subsequently taken up by the cell-bound Pst transport system [22]. The latter also supports the above-mentioned evidence that PstS and DING proteins coexist in some *Pseudomonas* strains, which they confer a highly adhesive and also virulent phenotype.

A lactoferrin-binding protein of 40 kDa from *Prevotella nigrescens,* a bacterium responsible of gingival diseases, has been purified from the outer membrane and shown to possess the conserved DING N terminus [1,23]. Other transferrin- or lactoferrin-binding proteins in the range of 38–42 kDa that could belong to the DING family have been described without being formally identified by sequencing [24].

Another small DING protein has been identified in *Bacillus mojavensis A21*; it is an alkaline serine-protease, with an estimated molecular mass of 20 kDa, highly active and stable in a broad pH range and to a wide range of commercial detergents [13] (Table 1). It is a secreted protein, with optimum temperature of 60 °C, and its N-terminal amino acid sequence _1_DINGGGATLPQKLYQTSGVL_20_, highly conserved in others annotated DING proteins [13] and with low identity with bacterial peptidases.

More recently a DING protein has been purified from the probiotic lactic acid bacterium *Pediococcus acidilactici,* as an extracellular caseinolytic alkaline enzyme [25] (Table 1). It is about 39 kDa and shares >90 % sequence similarity with the DING protein family [25].

As seen, bacterial DING proteins are similar to PstS proteins, which are ubiquitous in bacteria, but as some *Pseudomonas* ones, they are much more similar to the eukaryotic DING proteins.

### 1.2. DING Proteins in Eukarya

In Eukarya, DING proteins have been identified in animals (human, monkey, rat, turkey, etc.), plants (*Hypericum perforatum*, *Arabidopsis thaliana*, potato, tobacco, etc.), and fungi (*Candida albicans*, *Ganoderma lucidum,* etc.) [15,26] mostly as polypeptides of 40 kDa or higher [7]. In particular, DING proteins are present as isoforms of various molecular masses in mice. Their intracellular localization is tissue-dependent, being exclusively nuclear in neurons, but cytoplasmic and nuclear in other tissues [27]. Some of these proteins seem to have key roles in various human diseases, e.g., rheumatoid arthritis, atherosclerosis, and HIV suppression. Although this protein family seems to be ubiquitous in eukaryotes, their genes are consistently lacking from genomic databases [1].

The only eukaryotic DING protein with a complete amino acid sequence is the human phosphate binding protein (HPBP). It is a serendipitously discovered plasma apolipoprotein that binds phosphate and has been isolated from human plasma [28]. It forms hetero-oligomers that stabilize paraoxonase 1 (PON1), a plasma enzyme known for its antiatherogenic properties [29]; HPBP could thus be indirectly involved in protection against atherosclerosis [30,31] (Table 1).

The solved HPBP structure [16] has confirmed homology with bacterial PstS; it corresponds to a “Venus flytrap” model, in which two globular domains hinge together to create a phosphate-binding site between them, comprising the eight well conserved amino acid residues [15].

Three others human DING proteins involve the crystal adhesion inhibitor (CAI), the human synovial stimulatory protein (SSP), and the X-DING-CD4^+^ from human CD4^+^ T lymphocytes [35].

The crystal adhesion inhibitor (CAI), found in monkey and in human renal epithelial cells, has shown the property to bind calcium oxalate and to inhibit the growth of kidney stones [36].

In addition, the synovial stimulatory protein (SSP) is a DING polypeptide isolated from the synovial fluid of rheumatoid arthritis patients but not from that of healthy people, showing the capacity to induce proliferation of the peripheral blood T cells in patients with rheumatoid arthritis [1,37,38,39]. Another DING protein, the steroidogenesis-inducing protein (SIP) has mitogenic activity toward the ovarian epithelium and might be involved in the etiology of ovarian cancer [40].

X-DING-CD4 from CD4^+^ T cells inhibit HIV-1 replication through blockage of the LTR transcriptional activity [35,41], by inhibiting NF-kB binding to LTR [3].

It seems likely that eukaryotic DING proteins are secreted and then modified by N-terminal proteolysis. In fact the 39 kDa synovial DING protein was first identified as the proteolytic product of a 70 kDa monomer and larger precursors were immunochemically identified for other DING proteins [2].

**Figure 1:**
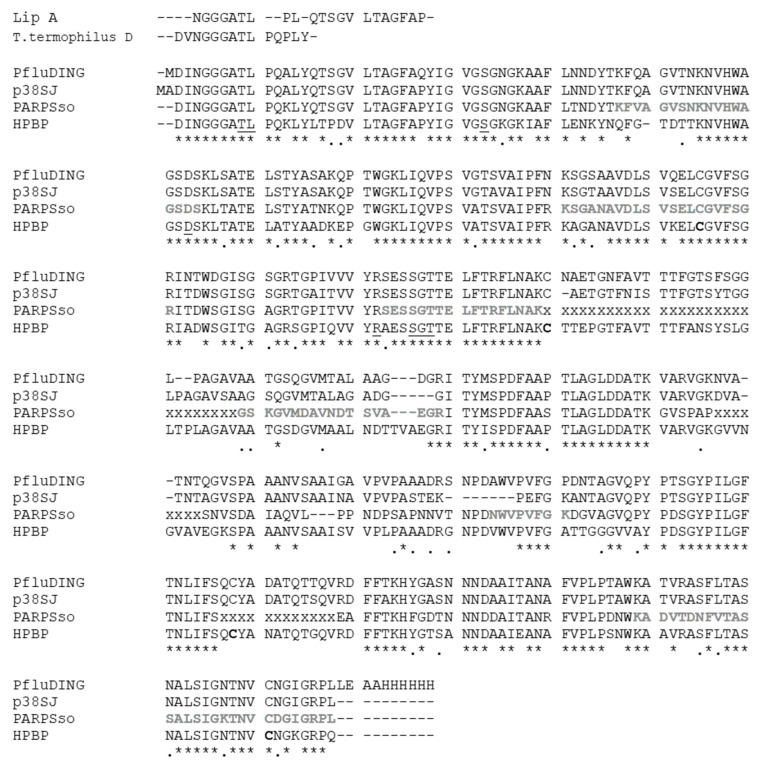
The amino acid alignments of representative mesophilic (HPBP, PfluDING, p38SJ, or pDING), thermophilic (*Thermus termophilus* DING, Lip A), and hyperthermophilic (PARPSso) DING proteins. Structural alignment was done by using the tools available in *Swiss PDB Viewer*software, and manually refined. The residues known in HPBP to be involved in phosphate binding are underlined, and conserved in all of them; cysteines forming disulfide bridges in HPBP are in bold; new sequenced part of PARPSso [47], not yet submitted, are shown in bold and gray; lacking parts of the PARPSso sequence are indicated with “x”; “*” and “.” mean identity and similarity respectively.

In plants various DING proteins have been fortuitously discovered during past years [2]; in particular the effect of inhibition of proliferation of human cell line by whole extract of callus culture of plant St John’s wort (*Hypericum perforatum*) let to the identification of a DING protein of predicted mass of 38 kDa [42]. The effort to clone the entire protein let to obtain a clone that produced a C-terminal truncated 27 kDa DING protein and was named p27SJ [26]. p27 was found to dramatically reduce HIV replication in astrocytes, by interacting with the endogenous transcription factor C/EBPb and the essential HIV transactivator protein (Tat), which leads to their subsequent colocalization to the cytoplasm where they are unable to influence transcription [26,43]. Later, a complete DNA coding sequence for a human DING protein, called p38SJ and than pDING, was deposited and corresponds to the full-length DING gene obtained from *H. perforatum* [2], whose effect on cell proliferation has been tested, in particular in suppressing proliferation of malignant glioma cells, interfering with cell cycle progression and several kinases that control cell proliferation of these tumors [44] (Table 1). We considered the original full-length protein p38SJ for alignment in Figure 1.

Recently, a 39 kDa DING-soluble cytoplasmic protein from leaves and seeds of the *Capsicum chinense* Jacq plant has shown, in addition to phosphatase activity, the ability to inhibit the growth of several plant and human pathogenic bacteria, including *Xanthomonas campestris, Erwinia carotovora, P. syringae, P. aeruginosa, Shigella flexnerii,* and *Staphylococcus aureus* [45], and also the growth of *Saccharomyces cerevisiae* and some cancer cell lines [12].

In *Candida albicans* a protein recognized as putative Complement receptor 3-related protein (CR3-RP) has been identified and seems to play a role in adherence of *C. albicans* to buccal epithelial cells, and in biofilm formation [46].

Although the amount of data concerning this protein family has increased over the last few years, their physiological functions remain largely unknown, and their origin in eukaryotes is still under debate [1].

### 1.3. DING Proteins in Thermophilic Organisms and Hyperthermophilic Archaea

Although many DING proteins have been identified in eukaryotic and bacterial organisms, few examples come from the extremophilic world. Quite recently in the thermophilic bacteria *Thermus thermophilus* it has been identified a phosphatase with a typical DING N-terminal sequence that interacts with the cell membrane [32]. It has a molecular mass of 40 kDa, and exhibits optimal phosphatase activity at pH 12.3 and 70 °C. This DING protein is a multifunctional enzyme with ATPase, endonuclease, and 3′-phosphodiesterase activities; moreover, it binds to linear dsDNA, displaying helicase activities and could thus be involved in DNA repair [32] (Table 1).

*T. thermophilus* DING protein isolated from the cytoplasm lacked its signal peptide present in other homologs, such as the DING protein from *P. aeruginosa* L-AP [48], indicating that the DING protein transport from the periplasmic space towards the cytoplasm may take place after truncation of its signal peptide. Moreover, its activation by oleic acid could be attributed to a hydrophobic origin of the enzyme that may initially be localized in the periplasm, or to its stabilization at high temperatures. Periplasmic expression of DING proteins appears to be related to signaling components that can interact with signal molecules only after passing through the bacterial outer membrane [21].

It is intriguing the discovery of a 50 kDa protein from the thermophilic bacterium *Thermosynthropha lipolitica* exhibiting maximal lipase activity at 96 °C [33]. “Lip A”is an extracellular lipase with high activity on long-chain fatty acid glycerides; attempts to clone this lipase were unsuccessful [33]. The thermostable lipase has N-terminal sequence “NGGGATLPLQTSGVLTAGFAP” that clearly identify it as a DING protein (Table 1).

In the hyperthermophilic archaeon *Saccharolobus solfataricus* a peculiar DING protein has been found, called PARPSso; it has been biochemically characterized as a poly-ADP-ribose polymerase-like enzyme (PARP-like) of 46.5 kDa [49], but its partially known sequence has been found to overlap those of DING proteins [8]. It has the characteristic DING N-terminal signature and represents the first member of the family isolated from Archaea: this finding extends the existence of DING proteins into all the biological domains of life [8]. It is worth noting that PARPSso tested with anti-PARP-1 antibodies gave a single immunosignal at 46.5 kDa, but under certain purification and treatment conditions, it converted into an immunoband with a nearly double molecular mass [50]. It has been previously demonstrated that both electrophoretic bands correspond to PARPSso and that the same anti-PARP cross-reactivity is exhibited by the eukaryotic HPBP and PfluDING [9] (Table 1). The PARP-like activity, measured for PARPSso, is rather unusual, since the previously characterized PARPs (a protein family of 18 members), share a highly conserved catalytic site not detected in the available partial amino acid sequence of the *Saccharolobus* thermophilic enzyme PARPSso [8,51]. In eukaryotes, the synthesis of poly(ADP-ribose) from NAD^+^ is catalyzed by the family of PARPs and acts as a regulatory mechanism of several nuclear and cell processes, depending on the PARP enzyme involved [51]. The *Saccharolobus* DING is membrane bound; it is localized along the periphery of the cell, although it is strictly associated with DNA suggesting a possible involvement in nucleic acid metabolic processes [34]. Moreover, it was recently reported that PARPSso has also ATPase activity, with a specificity for ATP even higher than for NAD^+^ [14]. This feature supports that, more than in other microorganisms, in the Archaea it is very common that proteins have more than one function. PARPSso shares the same activities with other members of the DING group, at different extents (Table 1).

## 2. Sequences and Structure

DING proteins are characterized by a highly identical DINGGG-amino acid sequence at the N-terminus and by unusually high sequence conservation, even between distantly related species. Only recently the large number of novel members of the family (over sixty), and their available amino acid sequences at N-termini allowed one to classify them in five subfamilies, often including both Prokaryotes and Eukaryotes in the same subfamily [2]. In particular, the longest sequence N-terminus (34 residues) was considered for comparison. In each subfamily amino acid identities are close or even higher than 90%, suggesting a high conservation in such different organisms [2].

In some proteins the availability of other sequenced peptides extends the identity further [2].

Untilnow the complete or extended amino acid sequences are known for a few DING proteins. Figure 1 shows sequence alignments of the most known ones from some mesophiles and thermo-hyperthermophiles. All these proteins share a high sequence identity. The most studied members, HPBP and PfluDING, have 71% identity; lower, but very close is the identity of the hyperthermophilic PARPSso, sequenced by 85% of its length. It is worth noting that the thermozyme aligns for more than 80% with p38^SJ^ protein (pDING) from *H. perforatum*, (St. John’s plant), and that both belong to the SBW3 subfamily [2].

The two mentioned thermophilic DING proteins, *T. thermophilus* DING and Lip A from *T. lipolytica* cannot be attributed to a given subfamily or group because they are known only from a limited number of N-terminal aminoacids (typically less than 15) [2] and therefore has been added manually for comparison (Figure 1).

The high sequence similarity of DING proteins accounts for the presence of conserved domains such as the site of phosphate binding. The ability of HPBP and PfluDING to bind phosphate ion (P) was largely demonstrated [1,16]. P is tightly bound by 12 hydrogen bonds with eight residues. These amino acids are highly conserved and were found in all DING proteins that were mostly sequenced (Figure 1). In PARPSso the eight conserved residues of the phosphate-binding loop (P-loop) are T8, L9, S32, D62, R141, S145, G146, and T147 [15], which correspond to the conserved eight ones in human and Pflu proteins.

The hydrogen bond with the aspartic acid was indicated as playing a key role in phosphate specificity by accepting protonated phosphate species [1].

The remaining percentage of sequences different among the studied DINGs might account for the variety of their specific properties. For instance HPBP is strongly hydrophobic, with large non-charged areas, whereas PfluDING has frequently charged regions [1,16]. This difference could allow one to explain their different solubility.

Structural studies mainly of HPBP and PfluDING revealed interesting details about their organization [1,16]. The availability of their three-dimensional structures confirmed that, similarly to the PstS, these DING proteins are formed by two elongated globular domains linked together by a flexible hinge allowing a “Venus flytrap” arrangement [15]. Each domain has a central beta-sheet and, around it, alfa-helices; the domain contains two disulphide bridges. The two globular domains are interfaced by an antiparallel two-stranded beta sheet forming the hinge and determining the cleft where the phosphate is buried. Further studies on PfluDING allowed to hypothesize that the ability of the protein to bind larger molecules rather than a single phosphate might be due to the induction of large conformational changes [1]. In particular, the changes from an “open” to a “close” conformation depend on the binding of a ligand.

Compared to PstS, DINGs represent an independent class of proteins because of the structural differences on four external loops protruding from the globular domains and for the presence of two disulfide bridges missing in PstS [1,15]. The four cysteine residues are conserved in DING sequences. In PARPSso the secondary structure predicted for the region involved in the globular domains indicates the beta-sheets flanked by alfa-helices described for other DING proteins; moreover, in the partially sequenced tracts, the presence of two invariant cysteines was observed [14].

This is in line with the structural studies reported by Sachdeva et al. (2013) in which four DINGs (from human, plant, and bacteria) show highly-conserved protein backbones and differ in the length of the protuberant loops [3]. The comparison we do with PARPSso, the thermostable DING from *S. solfataricus* (Figure 1), highlights that part of one of these loops (from 230 to 240 of PfluDING protein sequence) is still unknown for the extremophilic DING and quite variable between the other aligned proteins. We can hypothesize that this loop could be shorter respect to others mesophilic DINGs and usual in extremophilic proteins where generally shorter loops confer more stability to the protein [52].

The differences in the length of protruding loops could be related to the various protein/protein interaction probably connected to the different physiological functions of DING proteins, proteins of an increasingly enigmatic family.

## 3. Which Is the Keystone of DING Proteins?

A“protein family”is defined as a group of proteins that share a common evolutionary origin, reflected by their related functions and similarities in sequence or structure. Protein families are often arranged into hierarchies, with proteins that share a common ancestor subdivided into smaller, more closely related groups. The terms superfamily (describing a large group of distantly related proteins) and subfamily (a smaller group of closely related ones) are sometimes used in this context. According to this EMBL-EBI definition, the presently known DING proteins belong to the superfamily of phosphate-binding proteins, as all possess a conserved phosphate-binding pocket. The group of DING proteins, originally defined as a family by Berna et al. [4], contains proteins that are heterogeneous for the wide diversity of their activities. Therefore the subsequent subdivision in five subfamily by Bernier [2] seems to better distribute DING proteins on the basis of aminoacid identities of particular residues [1,2,12,15,16,17,18] within the N- terminal region. The residues forming the phosphate-binding pocket are perfectly conserved and this represents a clear shared property for DING proteins that are able to bind phosphate.

However, DING proteins belonging to the same subfamily show different activities not always related to each other. This deserves more attention as data accumulate.

For most of the DING proteins different activities have been detected, mainly hydrolytic activities; in bacterial DINGs, phosphatase, phosphodiesterase, and nucleotidase activity have been found [32,48,53]. They have been associated mainly with phosphate scavenging [15,22,54,55], in particular in *P. aeruginosa* clinical isolates and other pathogenic bacteria, where DING proteins seem to be clearly related in adhesion and virulence as part of phosphate scavenging or sensing system [20,56].

In eukaryotes, DING proteins associated enzymatic activities include mainly phosphatase [10], cutinase [57], protease [58], and β-esterase [59]; most of them were related to a broad range of disorders and biological processes. Some have been associated with cell receptor function for binding ligands ranging from simple ions like phosphate, to polypeptides such as germin-like proteins [37,60].

In Archaea and thermophilic bacteria, e.g., *S. solfataricus, T. thermophilus,* and *T. lipolytica*, for some of them, alkaline phosphorolytic activity, DNA binding ability, and ATPase activity are shared [9,14,32] (Table 1).

Despite the different apparently not correlated features/functions of DING protein, the keystone of the DING world seems to be the phosphate binding site that, due to its conservation in DINGs from Archaea to Eukarya, can represent the common starting point for one ancestor of these proteins.

The ability to bind phosphate is a peculiar feature, common to all the presently known DINGs. Up to date, a complete and comparative enzymatic characterization is not available, to compare enzymatic activities between different DINGs. Generally, during evolution, new enzyme activities often originate from promiscuous secondary activities that have become important for fitness due to a change in the environment or a mutation. Mutations that make a promiscuous activity physiologically relevant can occur in the gene encoding the promiscuous enzyme itself, but can also occur elsewhere, resulting in increased expression of the enzyme or decreased competition between the native and novel substrates for the active site.

In addition to the binding of phosphate, interactions between DING proteins and other ligands were described [1,14].

Some DING proteins can also interact with other proteins or do self-association, e.g., in *Pseudomonas* they can form adhesive appendages [55]; the human HPBP binds to PON1 as a stability helper and for maintaining phosphotriesterase activity of PON1 [30]; in *S. solfataricus* the thermozyme PARPSso forms homodimers or complex with Sso7 protein [14].

It is possible that the first ability to bind phosphate ion has represented the starting point for the evolution of the capacity to bind other molecules (at the same active site) or acquire other “features” to interact/regulate other proteins.

## 4. Where Are the Missing DING Genes?

Another peculiar feature, common to many DINGs, is the absence of a nucleotide sequence coding these proteins, except for *Pseudomonas* PfluDING and for plant pDING [2].

Such genes absence, in particular in eukaryotes, makes it very difficult to explain why these proteins are easily found in mammals and plants [45]. In 2009 Berna et al. reportedthat less than ten nucleotide sequences encoding DING protein have been obtained from eukaryotes, but none of them encode a complete protein [1]. In prokaryotes, DING proteins were originally found in *Pseudomonas,* for which numerous complete sequences are available. Therefore it was suggested that the genetic problem was restricted to eukaryotes [2]. However, up to date, DING proteins have been identified in many other bacterial species (e.g., *Bacillus* sp. and *P. acidilactici*), including thermophilic bacteria (e.g., *T. thermophilus* and *T. lipolytica*) and also in hyperthermophilic Archaea (*S. solfataricus*). In none of the genome of these species a DING gene has been found, and this extends the genetic mystery also to prokaryotes. It has been originally proposed that the presence of DING proteins in eukaryotic samples could represent the consequence of experimental contamination, or of symbiotic or pathogenic associations between *Pseudomonas* sp. and eukaryotic hosts [15,54]. This hypothesis seems to be inconsistent with the existence of high-molecular-weight DING precursor proteins, in both plants and animal, for which there is no genetic element in bacterial genomes [7,27,61]

On the same line, the presence of DING proteins purified from thermophilic and hyperthermophilic organisms [8,32,49], characterized at high temperature, excludes the possibility of a cross contamination, and confirm the real existence of DINGs in other non-*Pseudomonas* bacterial species, and of course in Eukaryotes.

Although there would be at least four different DING protein genes in human, whose N-terminal sequences match those predicted by some DNA sequence in GenBank [2], there seems to be an intrinsic difficulty of obtaining experimentally nucleotide sequences for DING proteins, except from most of *Pseudomonas* sp.

Some of the hypotheses that have been postulated to explain the failure to isolate the nucleotide sequence encoding the DINGs include the formation of complex secondary mRNA structures that make difficult their sequencing and PCR amplification in addition to the GC richness of their putative genes as part of rapidly renaturing DNA [2,62].

Moreover the presence of short nucleotide sequences very far from each other in the genome, as observed by searching in the annotated genome of *S. solfataricus*, could let us to think about a possible protein precursor (already reported for some eukaryotic DINGs) that rapidly become the mature DING.

Considering almost 600 proposed inteins have been described, spanning all three domains of life [63], it is intriguing to think that a “protein splicing-like” mechanism for DING proteins may exist in some bacteria and Archaea.This aspect could be more deeply investigated to try to clarify this “apparent” lack of the DING genes.

## 5. Conclusions

The world of DING proteins is very complex and they represent more than merely components of a membrane phosphate transport system [2].

The activation of bacterial alkaline phosphatase biosynthesis upon phosphate starvation is a classic example of induced enzyme biosynthesis in bacteria [64], which involves transmembrane signaling regulated by the level of phosphate in the environment. For each of the various DING proteins in the organisms belonging to the three domains of life, Archaea, Bacteria, and Eukarya, the phosphate ion represents the clear element common to all of them, although they bind it with different affinity. This ability to bind this ion or phosphorylated molecules is probably the common functional feature at the basis of other different activities and functions that different DING proteins show.

In PARPSso, the DING protein from hyperthermophilic *S. solfataricus*, the presence of multiple activities, such as high ATPase activity and low phosphatase and ADP-ribosylation activity, indicates that these latter activities, probably promiscuous for PARPSso, have then become, during evolution, the main activity in proteins in superior organisms up to human.

It is very important to underline that some important structural determinants can be at the basis of improvement of function that occurs during evolution [65]. However, the lack of clearly identified genes in the genomes of organisms from Archaea to Eukarya, apart from the presence in some *Pseudomonas* sp., raises a question: why do DING proteins exist? A unique and clear answer cannot still be delineated, but their ubiquitous presence in all the domains of life gives at least an indication that their role/s, and/or activity/ies and/or function/s are important for more complicated processes in which they are involved in.

## Figures and Tables

**Table 1:** List of some DING proteins that have been purified and identified by protein sequencing.

Organism	Domain	Protein Name	Protein Sequence (NCBI accession N°)	Protein Structure (PDB ID)	Protein Features	Ref
**Mesophiles**						
*Homo sapiens*	Eukarya	HPBP	GI:194368556	2V3Q	apolipoprotein that binds phosphate forms hetero-oligomers that stabilize PON1 low PARP activity low phosphatase activity	[9,28,30]
*Pseudomonas fluorescens*	Bacteria	PfluDING	GI:388327120	2Q9T	no phosphatase activity low phosphate affinity ATPase activity low PARP activity DNA binding capability	[9,21]
*Hypericum perforatum* (St John’s wort)	Eukarya	P27SJ P38SJ	AAW57408.1 AAW57408.2	n.s. n.s.	phosphatase activity interacting with the transcription factor C/EBPb and Tat	[26]
*Pediococcus acidilactici*	Bacteria	DING from Pediococcus	n.a.	n.s	extracellular Caseinolytic/proteinase activity	[25]
*Bacillus mojavensis* *A21*	Bacteria	A21 protease	n.a.	n.s.	secreted alkaline serine -protease optimal T at 60 °C	[13]
**Thermophiles**						
*Thermus thermophilus*	Bacteria	*T. thermophilus* DING	n.a.	n.s.	cytoplasmatic alkaline phosphatase ATPase/endonuclease/3′-phosphodiesterase activity linear dsDNA binding capability	[32]
*Thermosyntropha lipolytica*	Bacteria	LipA	n.a.	n.s.	extracellular alkaline lipase	[33]
**Hyperthermophiles/** **extremophiles**						
*Saccharolobus solfataricus*	Archaea	PARPSso	B3EWG9.1	n.s.	intracellular PARP activity low phosphatase activity membrane-binding ATPase activity DNA-binding ability	[8,9,14,34]

n.a.: not available; n.s.: not solved.

## Data Availability

Not applicable.
